# *phoP* maintains the environmental persistence and virulence of pathogenic bacteria in mechanically stressed desiccated droplets

**DOI:** 10.1016/j.isci.2023.106580

**Published:** 2023-04-08

**Authors:** Vishnu Hariharan, Atish Roy Chowdhury, Srinivas Rao S, Dipshikha Chakravortty, Saptarshi Basu

**Affiliations:** 1 Department of Mechanical Engineering, Indian Institute of Science, Bangalore, Karnataka State 560012, India; 2 Department of Microbiology & Cell Biology, Indian Institute of Science, Bangalore, Karnataka State 560012, India; 3 Interdisciplinary Centre for Energy Research (ICER), Indian Institute of Science, Bangalore, Karnataka State 560012, India; 4 Indian Institute of Science Education and Research, Thiruvananthapuram, Kerala State 695551, India

**Keywords:** Applied physics, Microbiology, Biophysics

## Abstract

Despite extensive studies on kinematic features of impacting drops, the effect of mechanical stress on desiccated bacteria-laden droplets remains unexplored. In the present study, we unveiled the consequences of the impaction of bacteria-laden droplets on solid surfaces and their subsequent desiccation on the virulence of an enteropathogen *Salmonella* typhimurium (STM). The methodology elucidated the deformation, cell-cell interactions, adhesion energy, and roughness in bacteria induced by impact velocity and low moisture because of evaporation. *Salmonella* retrieved from the dried droplets were used to understand fomite-mediated pathogenesis. The impact velocity-induced mechanical stress deteriorated the *in vitro* viability of *Salmonella*. Of interest, an uninterrupted bacterial proliferation was observed in macrophages at higher mechanical stress. Wild-type *Salmonella* under mechanical stress induced the expression of *phoP* whereas infecting macrophages. The inability of STM *ΔphoP* to grow in nutrient-rich dried droplets signifies the role of *phoP* in sensing the mechanical stress and maintaining the virulence of *Salmonella*.

## Highlights

• Bacterial pathogens can persist in mechanically-stressed desiccated droplets
• Bacterial cell walls deform flexibly under velocity-induced mechanical stresses
• Impact velocity of bacteria laden-droplets could not hamper its virulence
• Deleting *phoP* reduced bacterial survival in mechanically stressed dried droplets

## Graphical abstract

**Figure:**
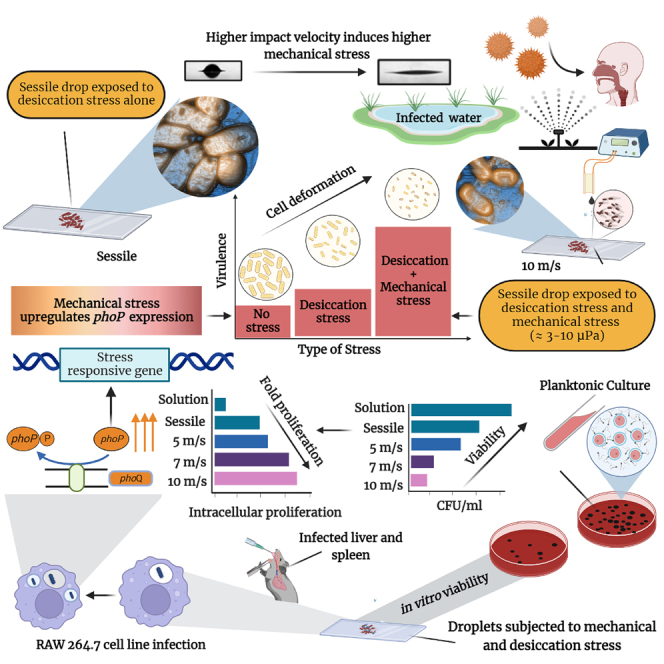

## Introduction

The bacterial droplets settling over inanimate surfaces are one of the leading causes of dissemination of microbial infection.^1^^,^^2^ The bioaerosols generated from contaminated water supply (like busted pipes), public restrooms, and wastewater treatment plants can be hotbeds to transmit airborne microbial infection.^3^ The bacteria-laden droplets from a contaminated water source or respiratory event of an infected person (cough, sneeze) usually impinge on various substrates (fomites) in the vicinity with specific velocities (kinetic energy). Such impact on substrates leads to severe deformations of the droplets because of significant mechanical stress inside the liquid phase. The bacteria inside the droplets experience these stress conditions for short durations (milliseconds). Furthermore, these droplets on substrates undergo evaporation under ambient airflow conditions, thereby creating desiccation stress (long timescales) on the bacteria, which leads to unique spatiotemporal assemblies and subsequent non-uniform bacterial deposit patterns. Even though harsh environmental stress conditions and nutrient limitations can decrease the microbial count, the transmission and virulence of bacterial pathogens recovered from fomites or dried droplets remain unaltered.^4^^,^^5^ However, how the mechanical (impact velocity) and desiccation stress in evaporating dried droplets shape the environmental persistence and long-term virulence of bacteria remains unclear. In the current study, we aimed to understand the stress response mechanism of bacteria present in bacteria-laden drops, which were subjected to varying stress conditions, and studied fomite-mediated bacterial pathogenesis. We present a unique combination of fluid mechanics studies combined with biological insights (including animal infection studies) to understand how stress translates to virulence.

*Salmonella* typhimurium (STM), one of the leading causes of self-limiting gastroenteritis and diarrhea in humans,^6^^,^^7^ has been used as a model system in the present study. However, the proposed experimental methodology is suitable for investigating any naturally dried bacterial droplets. There is no conclusive evidence that *Salmonella* infection occurs from the surfaces of fomites or mechanically stressed desiccated droplets. *Salmonella* is a common bacterial pathogen that severely impacts various food production industries.^8^ Recently, *Salmonella* was detected in the batch of chocolate produced by Barry Callebaut, the biggest chocolate factory in the world, having an average annual production of 2.2 million tons. *Salmonella* infection spreads to healthy individuals through contaminated food and water and causes conditions such as salmonellosis, which includes fever, diarrhea, and stomach cramps in the infected host.^9^^,^^10^ However, airway transmission of different *Salmonella* serovars, such as Enteritidis, Typhimurium, and Agona, has also been documented.^11^^,^^12^^,^^13^ Aerosolized *Salmonella* colonizes within the trachea, ceca, liver, and spleen of poultry broilers.^14^^,^^15^ Improper sterilization of medical equipment and aerosol-mediated transmission from contaminated feces lead to infections and associated outbreaks of *Salmonella*.^16^^,^^17^

Despite poor *in vitro* survival because of nutrient limitation, *Salmonella* Typhimurium retrieved from inanimate surfaces showed hyper-virulence in RAW264.7 cells.^4^ The ability of *Salmonella* to survive on inanimate surfaces in the presence of low moisture and fewer nutrients poses a risk of contamination of consumable food items. It has been reported that thousands of people die worldwide from *Salmonella* systemic infections, such as invasive non-typhoidal salmonellosis, *Salmonella*-induced meningitis, and neurological abnormalities.^18^^,^^19^^,^^20^^,^^21^^,^^22^ Moreover, *Salmonella* remains the second biggest cause of food-borne disease and the leading cause of hospitalization and death in the US.^8^^,^^23^ All these aspects add to the importance of understanding the effect of velocity-induced mechanical stress and evaporation-induced desiccation stress on *Salmonella*-laden droplets.

Each bacterium within the medium can experience contact force when acquainted with any surface. These contact forces can either be compressive or tensile, depending on whether the bacterial cell wall pushes itself onto or pulls away from the surface. Furthermore, they experience compressive forces when exposed to open atmosphere, deep-sea water environments, or inside the host cells. Moreover, inhalation, contact, and ingestion of bacteria-laden droplets and aerosols from polluted water can endanger human health. The bioaerosols or microdroplets from the infected persons can be altered by ambient airflow convection and transported much farther than 1.83m.^24^ A recent study indicates that the evaporation of droplets can result in aerosols that can drive the transmission of SARS-CoV-2.^25^ The aerosols expelled by the infected person during exhalation, coughing, and sneezing can deposit over inanimate surfaces exhibiting a wide range of droplet sizes and velocity scales. It can also act as a source of microbial contamination. Irrespective of the droplet size and the surface it impinges, the transmission ability of bacteria-laden droplets or fomites can lead to bacterial colonization inside the host.^4^ The impact dynamics profoundly depend on the physicochemical characteristics of impact substrate and droplet properties. The liquid droplet impact on surfaces is vital in various natural processes. The sudden burst of pipelines carrying sewage water and the velocity of droplets coming out from an infected person are some practical instances of drop impacts. These variations in impact velocities are characterized with the aid of several dimensionless numbers: The Reynolds number (Re = ρDV/μ), which is a dimensionless ratio of inertial to viscous force; Weber number (We = ρDV^2^/γ), which is the ratio of inertia to surface tension forces (γ), Ohnesorge number (Oh = μ/$\sqrt{\rho D\gamma }$), and Capillary number (Ca = μV/γ) where μ is the viscosity, ρ is the density, D is the diameter, γ is the surface tension forces, and V is the impact velocity. The impact mechanism for water drops at exceptionally low velocities was initially reported by Worthington.^26^ The literature reports substantial work on droplet spreading, receding, splashing, and bouncing.^27^^,^^28^^,^^29^^,^^30^

In the current study, the physiological relevance of bacterial droplet impact was studied by following an experimental protocol with impact velocities ranging from 5 to 10 m/s. The conditions considered mimic the velocity range of the droplets expelled from the mouth and nose during sneezing or coughing. The fluid dynamic events demonstrated in this study led to alterations in bacterial colonization, morphology, *in vitro* viability, subsequent infection pathways in macrophages, and bacterial gene regulations.

## Results

### Global impact dynamics of bacteria-laden droplets signifies the dominance of inertial effects

**Figure 1:**
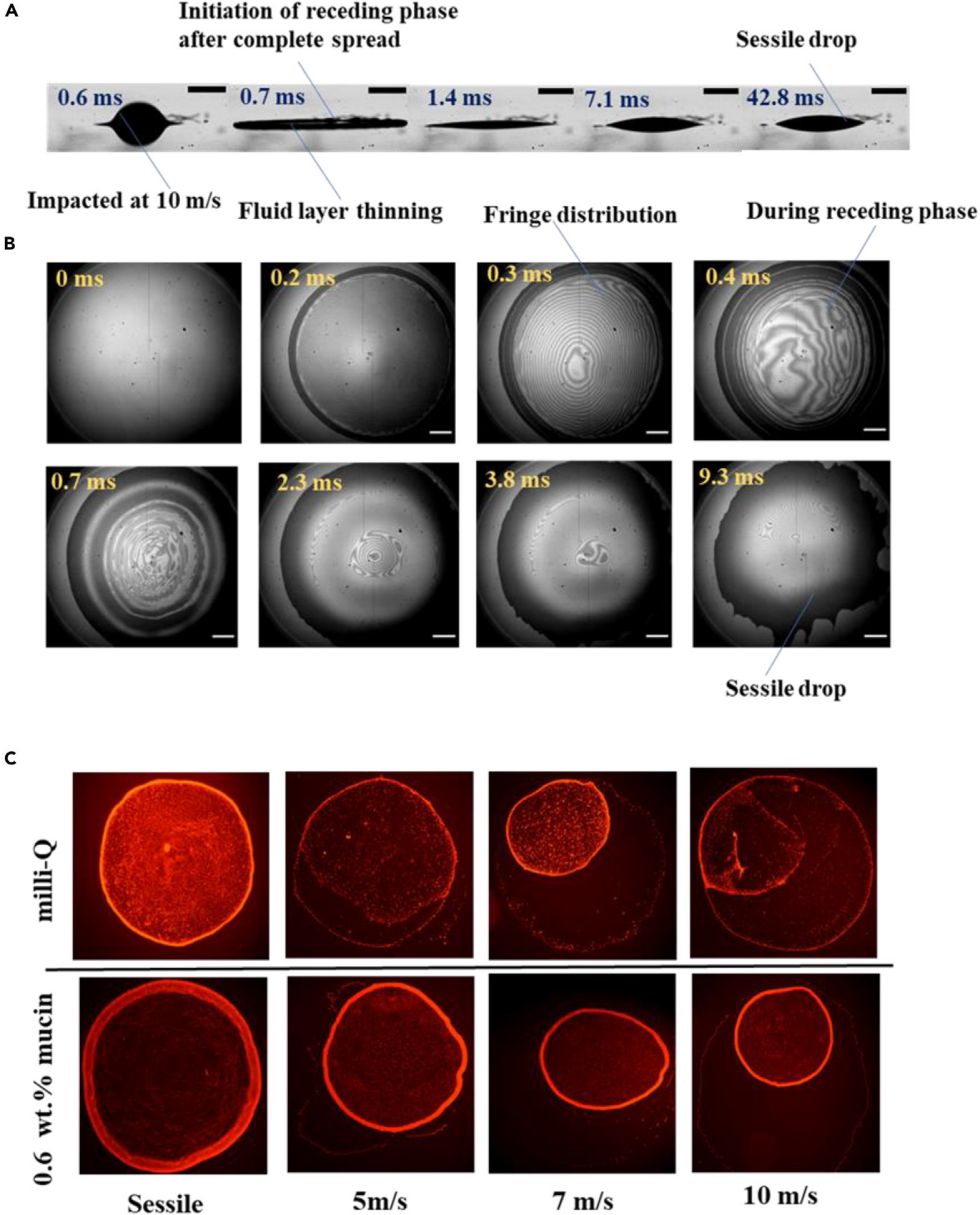
Global dynamics of bacterial droplet deposition (A) Profile view Images captured at 10000 fps for Salmonella laden droplet impacted on glass surface with 10 m/s on the glass substrate. The scale bar is 400 μm. (B) High-speed interference images captured at 10000 fps for Salmonella-laden droplet impacted on glass surface with 10 m/s on the glass substrate. The scale bar is 200 μm. (C) Fluorescence intensity captured for dried droplet impacted at different Weber numbers.

**Figure 2:**
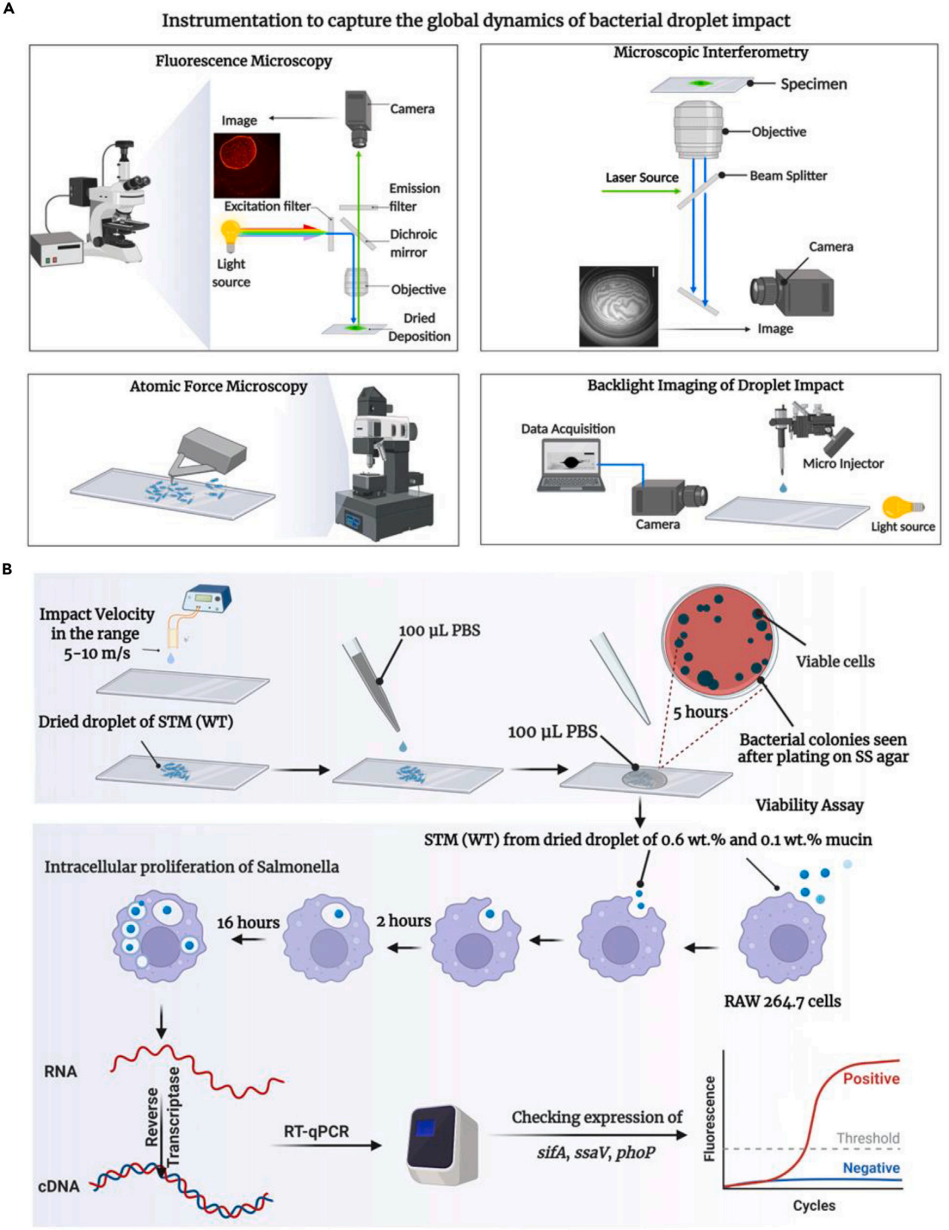
Schematic layout of the experimental study (A) The schematic representation to capture the global dynamics of droplet impact involving fluorescence microscopy, high-speed microscopic interferometry, atomic force microscopy, and backlight imaging of bacterial droplet impact. (B) Experimental layout depicting the assessment of *in vitro* viability of bacteria, infection of macrophages by the bacteria retrieved from desiccated dried droplets, and quantification of the expression of virulent genes of bacteria during infection.

The droplet spread over glass substrate primarily depends on the inertial effects, based on the Weber number corresponding to impact velocities of 5, 7, and 10 m/s leading to shorter timescale mechanical stress. The side-view imaging of the droplet impact on the glass surface is presented at various time instants (Figure 1A). Droplet impact sequence from the point of contact on the surface to large scale spreading and subsequent retraction leading to the formation of a sessile drop (i.e., for a higher velocity of 10 m/s) is evident from the snapshots. Figure 1B represents the spreading dynamics of the droplet at a higher impact velocity and the variation of thin-film liquid layers captured using reflection interference microscopy (Figure 2A). The droplet spreading is entrenched with various parameters such as viscous dissipation, surface tension effects, surface roughness, and contact line dynamics. These factors get overshadowed when the inertia forces are dominant. In this context, the present study demonstrates the influence of inertia forces rather than viscous effects during the bacterial droplet impact and spreading. The bacterial droplet spreads after the first point of contact on the surface and reaches a maximum spreading diameter (*d*_*max*_) until the initial kinetic energy is dissipated entirely. The spreading time also decreases with increased impact velocity because of the higher kinetic energy. Furthermore, when the drop attains *d*_*max,*_ the bacteria-laden drop retracts as the impact Weber number is significant, for which the maximum spreading diameter *d*_*max*_ is always greater than the equilibrium spreading diameter. Similarly, a higher receding velocity is observed for the higher impact velocity, as shown in Table 1. The interference patterns observed during the spread of the liquid layer signify the presence of a relatively thin liquid layer during the initial time instants for the maximum spread in the droplet. As time progress, retraction of the liquid layer is evident from the recorded images. Hence, the propagation of capillary waves of the receding liquid layer is seen as dark circular bands in the images from *t* = 0.3 to 0.7 ms (Figure 1B).

**Table 1:** Operating conditions on which the experiments are carried out indicate the impact velocities with the maximum spread and diameter of the drop investigated

Case number	Velocity (*m/s*)	Webernumber (-*We*)	Maximum spread factor *(-β)*	Average Receding velocity (*m/s*)
1	5.0 ± 0.3	100 ± 10	3.09	0.0171
2	7.0 ± 0.3	250 ± 10	3.74	0.0079
3	10.0 ± 0.3	750 ± 20	4.75	0.0054

The increase in multiple waves can be seen in the receding process of the liquid layer, and the disintegration of such waves is seen once it attains the shape of a sessile drop. The fringe patterns during the spread process are because of the thin-film liquid layer at lower droplet contact angles. The distribution of the fringe pattern relies on the increase in contact angle from the receding point to the formation of a sessile drop (Figure 1B). The extent of spread of the droplet is mainly dependent on the impact Weber number, and the spreading diameter and variation of the impact velocities are presented in Table 1. For all impact velocities, similar variations of the fringe patterns were evident from the reflection interference microscope. The fringe pattern during the thin film formation of the impacted droplet has been processed by employing fast-frequency guided sequential demodulation (FFSD) method. Fast-frequency guided algorithms are most suitable for transient, complex data and extraction of two-dimensional phase field to intricate fringe patterns.^31^^,^^32^^,^^33^^,^^34^ During the initial period, the spread of the thin film is symmetric as can be observed from even distribution of thin film thickness from the center of the droplet. The edge of the droplet spread is considered as the minimum film thickness (the reference point for measurement) as the glass surface is seen from the images (Figure 1B). With increase in time, the uneven distribution of the variation of film-thickness is observed from the fringe patterns and possible film-thickness (Figures S1A and S1B). A similar variation of film thickness is observed at the initial time instants and a reduction in thickness at higher time instants varying between 9 and 14 μm.

The global dynamics of droplet spread remained almost similar for various inertial forces, where the viscous measurements of working fluid are nearly similar to conventional fluids, i.e., distilled water (Figures S2A and S2B). The side-view and interference microscopy imaging have been performed simultaneously with high-speed imaging systems as the impact, spreading, and retraction occur in rapid alternating regimes. The increase in impact velocity of the bacteria-laden droplet leads to a more extensive spread of the liquid layer, as presented in Table 1, because of the increase in inertial force. The impact energy of the droplet at different velocities and the increase in receding velocity assist in determining the unique bacterial deposition patterns and bacterial virulence.

The drying effects of bacteria-laden sessile drops after impact on the surface are inadequately addressed in the literature. The evaporation of impacted droplets leaves a different dried pattern on the glass substrate compared to sessile deposition. It is observed that the deposited pattern can range from a simple ring-like structure referred to as a coffee ring or multiple coffee rings with uniform deposition, depending on the shear force and flow characteristics. Correspondingly, the bacteria-laden fluids revealed various patterns on the glass substrate after the impacted drop evaporated (Figure 1C). A thick outer ring of particles was seen in the dried biological STM-mucin samples. For a sessile drop, the fluid flows radially outwards to replace the evaporated fluid at the base edge of the drop, maintaining a constant radius thus leading to the commonly seen coffee ring.^35^^,^^36^ The pinning of the contact line ensures outward capillary flow that helps in carrying the material toward the edge of the pinned surface, as represented in the literature.^37^^,^^38^^,^^39^^,^^40^ Moreover, capillary flow creates a thick outer ring at its maximum spread when the droplet impinges at a certain velocity for which the viscosity of the base fluid medium is accountable for transporting particles toward the contact line.^41^ During the spreading phase, the change in contact line or angle variation mainly depends on the liquid and the surface properties. Furthermore, when the droplet recedes after reaching maximum spread, it experiences a constant contact angle regime, and the bacterial deposition was found to be uniform inside the ring. The variation in deposition patterns primarily indicates differences in bacterial morphology with an increase in impact velocities which is systematically investigated in the following sections.

The dependence of the bacterial residue on the impact velocity can be addressed from the spread factor of the droplet with the time interval instants. The shape factor and non-dimensional time parameters were estimated using the correlations reported by Du et al.^42^ and Rioboo et al.^43^ as follows:

(Equation 1) $$\beta =\frac{D}{D_{0}};t^{*}=\frac{tv}{D_{0}}$$

Where, in Equation 1, *D*_*0*_ is the initial diameter of the droplet (m), *v* is the impact velocity (m/s), and the *t* is the spatial time interval from the time of impact to constant contact line formation of the droplet (Figure S2B).

### Varying stress conditions immensely altered the bacterial morphology

**Figure 3:**
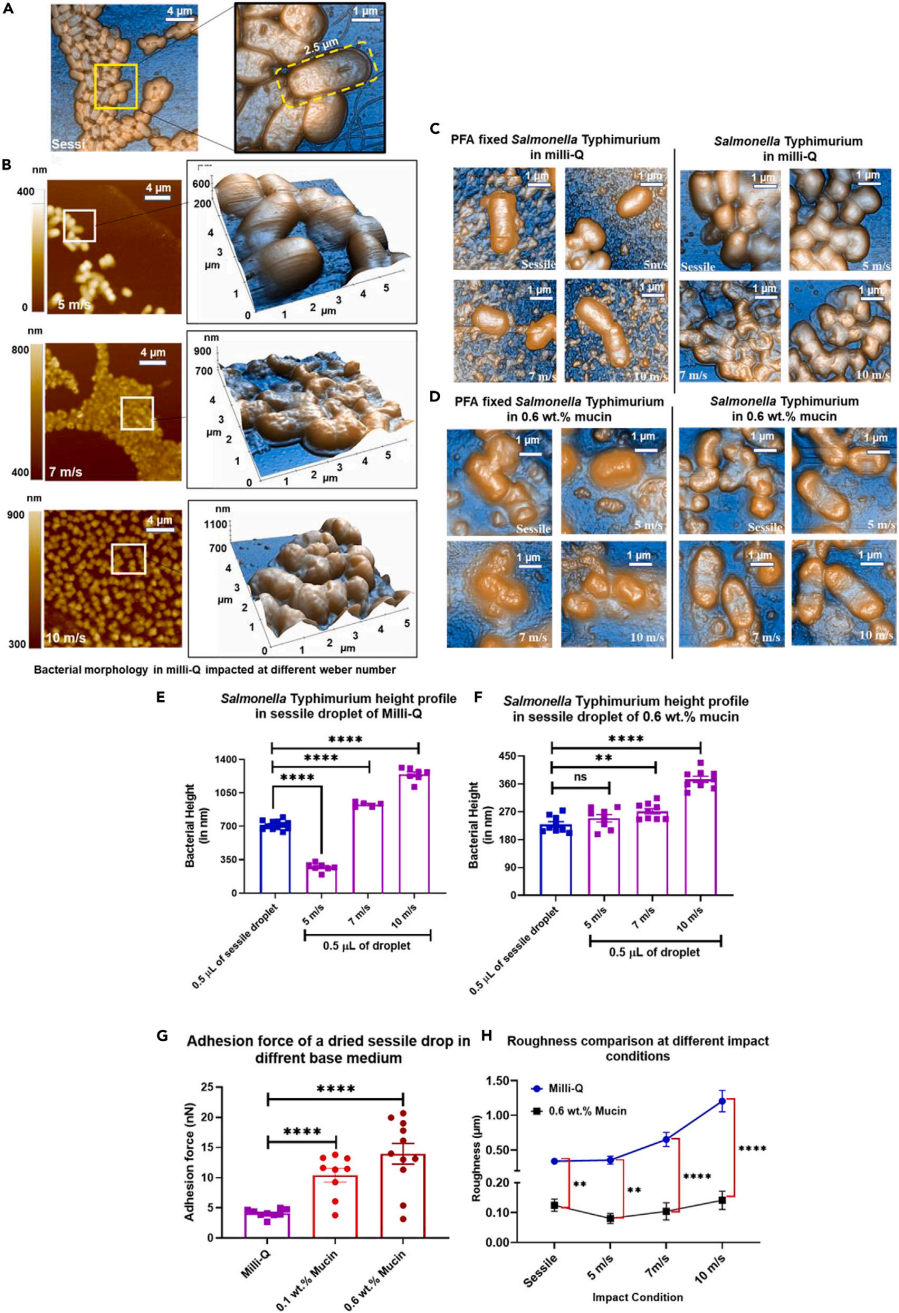
Atomic force microscopy identifies higher stress induction and greater surface damage at a higher impact velocity (A) AFM images for *Salmonella* in milli-Q in a sessile drop (B) AFM images of the bacteria-laden droplet with milli-Q as the base medium at three different impact velocities in a 5 μm square area. (C and D) (C) AFM images of the bacteria-laden droplet with milli-Q and (D) 0.6 wt % Mucin as the base medium at three different impact velocities in a 5 μm square area for PFA fixed cells. (E) AFM images of the bacterial-laden droplet with milli-Q and (F) 0.6 wt % mucin as a base medium compared with PFA fixed bacteria at three different impact velocities along with their height profiles in 5 μm square area. (G) Adhesion energy in nutrient-neutral (milli-Q) and nutrient-rich (0.6 wt % Mucin) base fluid medium experienced on a dried STM (WT) sessile drop. (H) Roughness measurements presented as RMS roughness at different impact conditions for milli-Q and 0.6 wt % mucins. *(P)* ∗<0.05, *(P)* ∗∗<0.005, *(P)* ∗∗∗<0.0005, *(P)* ∗∗∗∗<0.0001, ns= non-significant, (Student’s t test-unpaired).

From the discussions in the earlier section, inertial force is found to have a significant role in altering bacterial morphology. The topographic images of the bacterial cell wall at three impact Weber numbers (100, 250 and, 750) corresponding to 5, 7 and, 10 m/s are acquired using Atomic Force Microscopy (AFM) in nutrient-neutral (milli-Q) and nutrient-rich medium (0.6 wt % mucins). The coupling of mechanical forces with microscopic characterization is essential for comprehending physiological changes (Figures 2A and B). The qualitative analysis of the droplet impact with rapidly changing conditions significantly affects the surface morphology of the bacteria. Like other Gram-negative pathogens, *Salmonella* has a rigid cell wall made of peptidoglycan, a repeating unit of N-acetyl muramic acid and N-acetyl glucosamine connected by pentapeptide linkage.^44^^,^^45^ In response to environmental stimuli, *Salmonella* modifies its peptidoglycan layer.^46^ Also, when subjected to a certain velocity, the shape regulation of the *Salmonella* cell wall is profoundly affected by the external forces that affect the rate of cell wall synthesis.^47^ Apart from the impact stress, the bacterial cell wall is subjected to evaporative stress, which does not allow the bacteria to snap back to its initial cell wall shape (Figure 3A), as vividly observed for We = 250 (5 m/s) and 750 (7 m/s) for milli-Q and nutrient-rich medium^48^(Figures 3B–3D).

**Figure 4:**
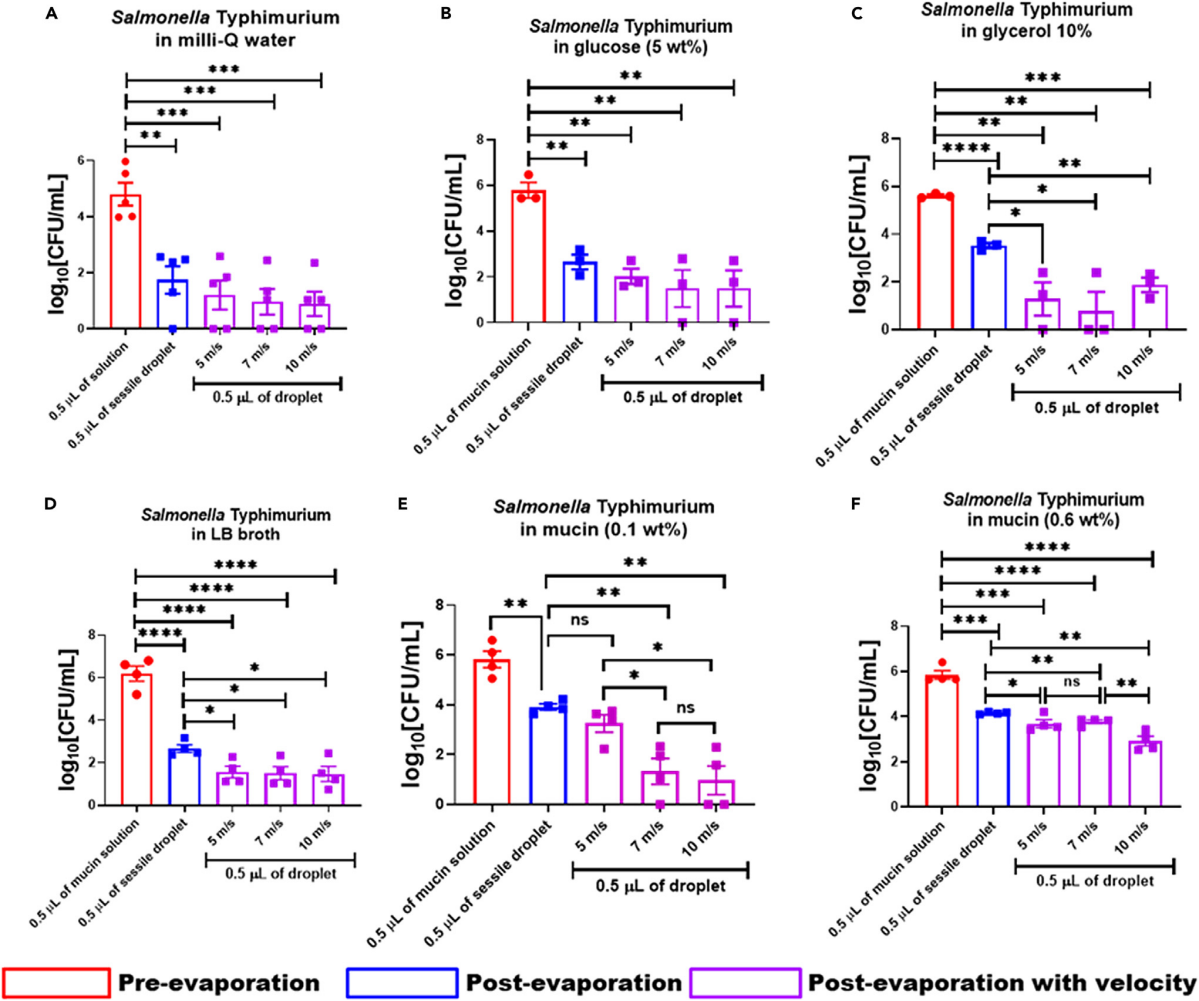
Assessment of *in vitro* viability of *Salmonella* retrieved from desiccated droplets of different base solutions impacted on a solid surface with or without mechanical stress induced by impact velocity The viability of *Salmonella* Typhimurium (in log10 scale) recovered from dried sessile droplets of different base solutions impacted on solid glass surface with or without velocities (5, 7, and 10 m/s). As base solutions. (A–F) milli-Q, (B) glucose 5 wt %, (C) 10% glycerol, (D) LB broth, (E and F) mucin 0.1 and 0.6 wt %, were used (N≥3). Data are represented as mean ± SEM. *(P)* ∗< 0.05, *(P)* ∗∗< 0.005, *(P)* ∗∗∗< 0.0005, *(P)* ∗∗∗∗< 0.0001, ns= non-significant, (Student’s t test-unpaired).

The maximum bacterial height of *Salmonella* when subjected to desiccation stress and the mechanical stress increased from 712.6 (sessile) to 1244.3 nm (10 m/s) in milli-Q medium (Figure 3E). A similar trend is seen when milli-Q is replaced with a nutrient-rich, 0.6 wt % mucin solution (Figure 4F), indicating flexible behavior of the cell walls (Figures 3C and 3D). AFM data also supports the view that the cell walls of *Salmonella* Typhimurium deform flexibly in the milli-Q and mucin medium (Figure 3C) because of nutrient deficiency and evaporative stress. Moreover, the flow shear stress is induced in the cell walls of the bacteria at higher impact velocities (Figures S3A and S3B). The height profile of a randomly selected bacterial cell (Figures S4A and S4B) indicates the presence of peaks and valleys over the cell wall. The deformation in the cell wall can be quantified using the roughness parameter.

The root means square roughness (*R*_*q*_) is calculated for *Salmonella* Typhimurium in milli-Q, and 0.6 wt % mucins are calculated based on the following Equation 2 for sessile and impact conditions (5, 7, and 10 m/s).

(Equation 2) $$R_{q}=\sqrt{\frac{1}{n}\sum_{k=1}^{n}z_{k}^{2}}$$

where *R*_*q*_ is the root-mean-square roughness at a height ‘*z’* for the pixel ‘*k*’ in the image. The *R*_*q*_ indicates the distance between peaks and valleys or the average height variations from the mean line. Physically, *R*_*q*_ demonstrates the extent of surface damage which is more significant in milli-Q than in 0.6 wt % mucins for Weber number greater than 250 (5 m/s) (Figure 3G). The stress experienced on the deposition surface, calculated from the force exerted by AFM probe, shows higher surface stress (Figures S3A and S3B) for a velocity greater than 7 m/s with increased surface roughness. The nano-intender used for AFM measures the adhesion energy for nutrient-rich and nutrient-neutral samples by quantifying the forces between the tip and the sample.

The force-displacement spectroscopy for all the samples indicates that the force experienced during approach and retraction of the probe over the dried bacterial sample is significantly different, depicting greater adhesion between the tip and the cell walls, especially for velocity greater than 7 m/s (Figure 3H). Furthermore, higher adhesion energy indicates enhanced interaction of cells with the adjacent cells and the substrate. The enhanced cell-cell interaction leads to local stiffening of the dried deposit resulting in variations in the adhesion energy and resisting the deformation caused because of the receding velocity *(dβ/dt)* at different impact conditions. This enhanced interaction between the cells and substrate cushions the bacteria, keeping it viable even in adverse conditions.^49^ The increased surface roughness has a pertinent role in microbial adhesion.^50^^,^^51^ The undulations on the surface of bacterial deposits caused because of surface roughness enhanced their adhesion on glass substrate because of increased surface area and fissures at higher impact velocities. Moreover, cell-cell interaction or cushioning of bacteria happens when the adhesion energy is large, especially in the case of nutrient-rich 0.6 wt % mucins compared to a nutrient-deficient milli-Q medium (Figure 3G).

The cushioning effect on the cell membranes is quantitatively assessed using roughness kurtosis. The roughness kurtosis (*R*_*ku*_), acquired from AFM, provides the sharpness of spikes on the surface, which physically denotes that a grooved or pitted surface could shelter the bacteria by providing a cushioning effect. The numerical value of *R*_*ku*_ greater than three indicates a presence of a spiky surface, and *R*_*ku*_ less than three denotes a bumpy surface^52^ given by Equation 3.

(Equation 3) $$R_{ku}=\frac{1}{nR_{q}^{4}}\sum_{k=1}^{n}z_{k}^{4}$$

where *R*_*q*_ is the root-mean-square roughness at a height ‘*z’* for the pixel ‘*k*’ in the image. The average roughness kurtosis is ≈5.1 in the case of bacteria-laden 0.6 wt % mucins, ≈4.2 in 0.1 wt % mucins, and ≈1.1 in milli-Q sessile drop. The spiky and bumpy surfaces are evident from the AFM images (Figures 3A, 3C, and 3D). This indicates that mucin (0.1 and 0.6 wt %) provides a better cushioning effect to the bacteria trapped between the spiky surfaces than milli-Q and influences the *in vitro* viability of the bacteria. However, the viability of the bacteria is also subjected to nutrient availability in the medium, which requires further investigation, as discussed in the subsequent sections.

### The impact velocity reduced the *in vitro* viability of *Salmonella* Typhimurium in nutrient-rich desiccated droplets

Earlier, we reported that irrespective of the presence of nutrients, the *in vitro* viability of wild-type *Salmonella* inside the sessile droplets is severely compromised post-evaporation. Moreover, the bacteria recovered from dried nutrient-rich sessile droplets hyper-proliferates inside the RAW264.7 macrophages.^4^ However, the effect of mechanical stress because of the impact velocities of the droplets on the *in vitro* viability of the bacteria has not been investigated before. In this study, we have used neutral (milli-Q), low-nutrient (glucose 5 wt %, and 10% glycerol), and nutrient-rich (LB broth, mucin 0.1 wt %, and 0.6 wt %) base solutions for impacting the bacterial droplet on the glass surface with specific velocities (5, 7, and 10 m/s) as mentioned earlier. 0.5 μL of the planktonic culture of wild-type *Salmonella* in different base solutions gave rise to 10^5^ to 10^6^ CFU of viable bacteria (Figures 4A–4F; red bar). In the dried sessile droplet of 0.5 μL volume, the viability of STM (WT) reduced drastically to 10^2^ from 10^4^ CFU (Figures 4A–4F; blue bar). The loss of moisture because of evaporation restricted the availability of nutrients to the bacteria in the dried droplet and decreased the bacterial burden compared to the liquid-phase culture. In milli-Q (Figures 4A) and 5% glucose solutions (Figure 4B), the impact of droplets carrying wild-type *Salmonella* on the glass surface did not create any significant difference in its viability (∼10^2^ CFU) compared to the sessile droplet (∼10^2^ CFU). *Salmonella* can survive in glycerol by upregulating the genes associated with lipopolysaccharide biosynthesis and gluconate metabolism.^53^ When the bacteria were allowed to impact the glass slide with glycerol (10%) as the base medium (Figure 4C), we observed a significant decrease in the *in vitro* viability of *Salmonella* (∼10^2^ CFU) compared to the sessile droplet (∼10^4^ CFU) under all three velocities (Figure 4C). The reduction in the bacterial viability under velocity is attributed to mechanical stress induced by impact velocity (Figures S2A and S2B). Similar observations were obtained when bacteria-laden nutrient-rich-base solutions such as LB broth (Figure 4D), 0.1, and 0.6 wt % mucins (Figures 4E and 3F) were subjected to impact velocity. The viability of STM (WT) in the sessile droplet (∼10^3^ CFU) of LB broth was significantly reduced to ∼10^2^ CFU when exposed to mechanical stress (Figure 4D). A more prominent velocity-induced reduction of *in vitro* viability was observed in 0.1 wt % mucin solution (Figure 4E). Mucins are heavily glycosylated O-linked glycoproteins synthesized and secreted by the epithelial cells.^54^ The availability of nutrients in mucin improved the survival of the bacteria in the sessile droplet (∼10^4^ CFU) even when subjected to an impact velocity of 5 m/s (∼10^4^ CFU) velocity (Figure 4E). With an increase in the impact velocity from 5 to 7 m/s, the viability of wild-type *Salmonella* decreased from ∼10^4^ CFU to ∼10^2^ CFU and remained constant at 10 m/s (∼10^2^ CFU) (Figure 4E). Furthermore, when we used 0.6 wt % of mucin, we observed that the higher nutrient content overshadowed the effect of impact velocity on the *in vitro* survival of *Salmonella* (Figure 4F). Unlike 0.1 wt % mucin concentration, no significant change in the *in vitro* viability of the bacteria was observed at 5 and 7 m/s impact velocities (Figure 6F). However, the viability of STM (WT) reduced from ∼10^4^ CFU to ∼10^3^ CFU with an increase in the impact velocity from 7 to 10 m/s (Figure 6F). Collectively the present data suggest that in addition to desiccation and nutrient limitation stress, the mechanical stress induced by the impact velocity also reduces the bacterial burden in dried droplets post-evaporation.

### Wild-type *Salmonella* recovered from the desiccated droplet of mucin subjected to higher impact velocity showed hyperproliferation in RAW264.7 cells

**Figure 5:**
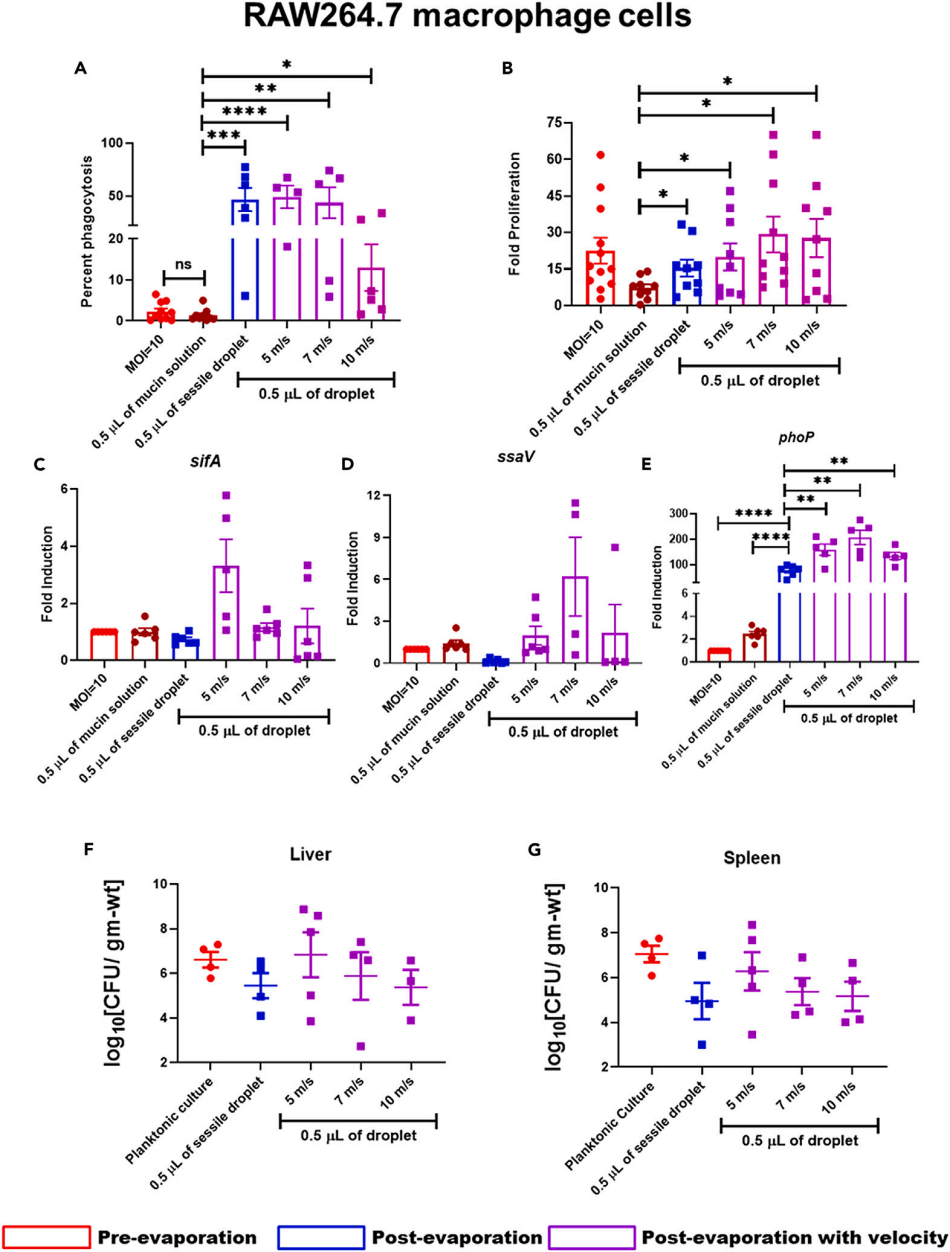
The virulence of *Salmonella* Typhimurium retrieved from desiccated mucin droplet (0.1 wt %) impacted on a solid surface at different velocities (A–G) *Salmonella* Typhimurium recovered from desiccated mucin (0.1 wt %) droplet impacted solid glass surface with or without specific velocities (5, 7, and 10 m/s) were used to infect RAW264.7 cells to determine the (A)percent phagocytosis and (B) intracellular proliferation (n = 3, N = 2). Determining the transcript-level expression of (C)*sifA,*(D)*ssaV,* and (E)*phoP* from intracellular *Salmonella* by RT-qPCR (n = 3, N = 2). *Salmonella* retrieved from the bacteria-laden dried droplet (mucin 0.1 wt %) impacted solid glass surface with or without velocities were used to infect C57BL/6 mice. On sixth day post-infection, the mice were sacrificed, and the bacterial burden in the (F) Liver and (G) Spleen was enumerated. Data are represented as mean ± SEM. *(P)* ∗< 0.05, *(P)* ∗∗< 0.005, *(P)* ∗∗∗< 0.0005, *(P)* ∗∗∗∗< 0.0001, ns= non-significant, (Student’s t test-unpaired).

We further investigated the effect of velocity-induced mechanical stress in the intracellular virulence of *Salmonella* retrieved from the dried droplet of mucin. After crossing the intestinal mucosal barrier, *Salmonella* interacts with phagocytic immune cells such as macrophages, dendritic cells, neutrophils, etc.^10^ Professional antigen-presenting cells like macrophages phagocytose invading pathogens thereby limiting their spread throughout the host body.^55^^,^^56^ Our study revealed that *Salmonella* recovered from dried sessile and velocity-impacted mucin (0.1 and 0.6 wt %) droplets are prone to phagocytosis by RAW264.7 cells compared to the planktonic culture (Figures 5A and S5A), suggesting a better clearance of the bacteria and subsequent restriction of the infection by macrophages. Surprisingly, these phagocytosed bacteria hyper-proliferated inside the macrophages (Figure 5B), indicating that the bacteria droplet, even when subjected to high mechanical stress because of impact velocity, can efficiently maintain their virulence while infecting the host cells. Wild-type *Salmonella* recovered from the velocity-impacted post-evaporated dried droplets of high mucin concentration (0.6 wt %) exhibited a similar phenomenon while infecting the RAW264.7 cells (Figure S5B). Inside the host cells, *Salmonella* stays inside a membrane-bound acidic compartment called *Salmonella*-containing vacuole (SCV).^57^ The acidic pH of SCV is sensed by the PhoQ/PhoP two-component system (TCS) of *Salmonella*, which uses *Salmonella* pathogenicity island-2 (SPI-2) encoded virulent factors such as *sifA*, *ssaV*, etc., for its successful replication inside the host cell.^58^^,^^59^^,^^60^ We hypothesized that the velocity-induced mechanical stress upregulated the expression of PhoQ/PhoP TCS and SPI-2 genes in *Salmonella*, which helped in the successful proliferation of the bacteria inside the macrophages. With an increase in the impact velocity (5–10 m/s) of *Salmonella* retrieved from 0.1 (Figures 4C–4E) and 0.6 wt % (Figures S5C–S5E) mucin droplets, showed an increased transcript-level expression of *sifA* (Figure 5C), *ssaV* (Figure 5D), and *phoP* (Figure 5E), which explains the reason behind their enhanced proliferation inside the macrophages. The uninterrupted proliferation and greater expression of *sifA*, *ssaV*, and *phoP* in intracellularly growing *Salmonella* further suggested intact *in vivo* pathogenesis. To validate this hypothesis, the animal infection study was carried out on C57BL/6 mice with *Salmonella* recovered from the bacteria-laden dried droplets (with or without impingement) and compared the bacterial burden of liver and spleen with the mice infected with a planktonic culture of *Salmonella* (Figures 5F and 5G). The comparable bacterial load of *Salmonella* retrieved from the bacteria-laden dried droplets (impacted the solid surface with or without velocity) in the liver and spleen of infected C57BL/6 mice suggested that the *in vivo* pathogenesis of *Salmonella* is not compromised due to mechanical and desiccation stress (Figures 5F and 5G).

### Survival of wild-type *Salmonella* in the velocity-impacted desiccated mucin droplets depends upon *phoP*

**Figure 6:**
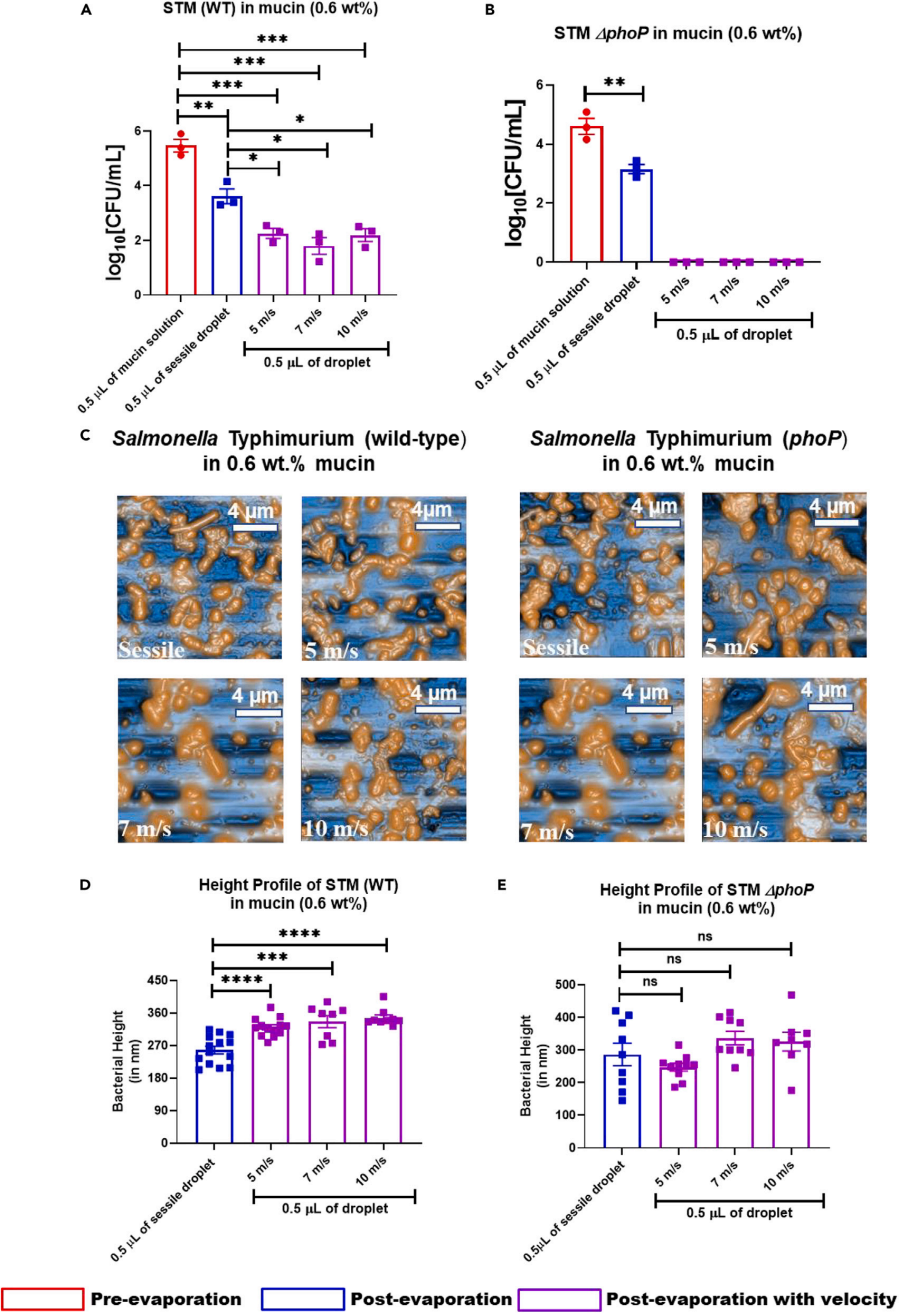
Deleting *phoP* reduced the *in vitro* viability of *Salmonella* Typhimurium in desiccated droplet of mucin (0.6 wt %) impacted on glass surface impacted with velocity 5, 7, and 10 m/s (A–E) The *in vitro* viability of (A) STM (WT) and (B)*ΔphoP* (log10 scale) recovered from dried sessile droplets of mucin (0.6 wt %) impacted solid glass surface with or without velocities (5, 7, and 10 m/s) (N≥3). (C) AFM images of bacteria (STM WT and *ΔphoP*) laden droplets in mucin (0.6 wt %) on the glass surface. Height profile of (D) STM (WT) and (E)*ΔphoP* from the desiccated droplets of mucin (0.6 wt %) on the glass surface. Data are represented as mean ± SEM. *(P)* ∗< 0.05, *(P)* ∗∗< 0.005, *(P)* ∗∗∗< 0.0005, *(P)* ∗∗∗∗< 0.0001, ns = non-significant, (Student’s t test-unpaired).

As wild-type *Salmonella* recovered from the dried droplet of mucins (for sessile and impact velocities 5, 7, and 10 m/s) indicated a consistent upregulation in the expression of *phoP* while infecting macrophages, we hypothesized that the survival of *Salmonella* Typhimurium in the nutrient restricted environment of dried velocity-impacted droplets depends on *phoP*. To test this hypothesis, the *in vitro* viability of STM (WT) (Figure 6A) and *ΔphoP* (Figure 6B) was measured in sessile droplets of high concentration mucin (0.6 wt %) to the impact Weber numbers of 100, 250, and 750. As discussed earlier, the viability of STM (WT) (∼10^6^ CFU–∼10^4^ CFU) and *ΔphoP* (∼10^5^ CFU–∼10^3^ CFU) was compromised in the sessile droplet of mucin after evaporation (Figures 6A and 5B). The bacteria-laden mucin droplets subjected to 5, 7, and 10 m/s velocities further reduced the viability of STM (WT) to ∼10^2^ CFU because of the generation of mechanical stress (Figure 6A). On the contrary, STM *ΔphoP* struggled to survive in the desiccated mucin droplets for impact Weber numbers of 100–750 (Figure 6B). The inability of STM *ΔphoP* to survive in the stressed environment of velocity-impacted desiccated droplets showed a novel role of *phoP* in assisting bacterial pathogens in tolerating mechanical stress. The deformation of the bacterial cell walls is an intrinsic aspect of impact velocity that affects the bacterial cell-cell interactions and surface topography. As demonstrated earlier, bacterial cell deformation depends on critical parameters such as impact velocity, evaporative stress, and adhesion energy. In the case of an impact velocity of 5 m/s, the stretched time scales delayed the deformation of the bacterial cell phase because of lower receding velocity (Table 1). Likewise, the increase in height profiles with an increase in the impact velocity results in enhanced squeezing of bacterial cell walls. Of interest, the height profile of STM (WT) (Figure 6D) was strikingly different from the STM *ΔphoP* (Figure 6E). The STM *ΔphoP* exhibited an unresponsive height variation for the impact velocity, 5 m/s, and the sessile drop (Figure 6E). Similarly, a negligible increase was noted for STM *ΔphoP* even when the velocity is ramped up to 7 and 10 m/s. This indicates that the change in height profiles of *Salmonella* in desiccated droplets depends profoundly on *phoP* when subjected to velocity-induced mechanical stress.

## Discussion

Earlier, multiple studies reported a reduction in the viability of bacterial pathogens when exposed to antibacterial surfaces.^61^^,^^62^^,^^63^ However, the present study demonstrated that investigating bacteria-laden drop impact on surfaces is highly relevant from the disease transmission perspective. This novel approach to droplet impact study opens a new dimension that brought out the effect of impact velocity-induced mechanical stress and evaporation-induced desiccation stress on the survival of bacteria in the nutrient-rich and nutrient-deficient medium. The spread of *Salmonella* from contaminated fomites or via aerosol is emerging as an important route of infections.^14^^,^^64^ Our study has discovered a few of the essential virulent genes of *Salmonella* that are required for their survival in desiccated droplets by bridging visible and microscopic perspectives. Even though the bacterial cell wall deformation is extensive at the higher impact velocity, the parameters such as receding velocity and adhesion energy also play an essential role in controlling the deformation. An increase in the bacterial cell height profile was noted for a higher impact Weber number because of high receding velocity occurring in smaller time scales. Compared to milli-Q, the enhanced adhesion energy in a nutrient-rich medium (0.1 and 0.6 wt % mucins) provides cushioning effect to the bacterial cell walls to resist the mechanical stress induced by the impact velocities. The better survival of wild-type *Salmonella* in the dried droplet of mucin (0.1 and 0.6 wt %) than in milli-Q, glucose, and glycerol indicated that the bacterial viability subjected to desiccation and mechanical stress also depends on nutrient availability. However, compared to the sessile droplets, the reduction in the bacterial viability on induction of velocity suggested a prominent role of mechanical stress in limiting the environmental persistence of pathogenic bacteria. *Salmonella* Typhimurium retrieved from the velocity-induced dried droplet showed intact intracellular proliferation within macrophages and C57BL/6 mice. Multiple studies showed that *Salmonella* requires PhoQ/PhoP two-component system to survive within the host cells.^65^^,^^66^^,^^67^ Compared to the macrophages infected with the planktonic culture of *Salmonella* (grown in LB and mucin solutions), the enhanced and consistent expression of *phoP* from the bacteria recovered from velocity-induced or uninduced desiccated droplets delineated the mystery behind their intact intra-host virulence. The inability of *phoP-*deficient *Salmonella* to grow in evaporated nutrient-rich base solution (mucin 0.6 wt %) with high impact velocity suggested the paramount importance of *phoP* in assisting *Salmonella* to sense mechanical stress. The bacteria-laden droplet impacting the surface with specific velocities significantly reduced the viability of bacteria during desiccation with the assistance of additional mechanical stress. Earlier our group reported that *Salmonella* surviving in the dried fomites could efficiently proliferate in the host macrophages.^4^ In the current study, we showed that even when subjected to high mechanical stress, the ability of the pathogen recovered from the dried fomites to cause successful infection is not hampered. To the best of our knowledge, our study proposed a novel and unique role of *phoP*, a two-component system gene of *Salmonella* Typhimurium, to sense the mechanical stress during the formation of dried fomites. Available reports suggest that many other pathogenic bacteria, such as *Acinetobacter baumannii*, *Pseudomonas aeruginosa*, and *Klebsiella pneumoniae,* can persist on inanimate solid surfaces in a nutrient-deprived environment for a very long time.^68^^,^^69^^,^^70^ These bacteria are often associated with hospital-acquired infections in humans. Our findings open a promising avenue for understanding the complex connection of naturally dried *Salmonella* droplets subjected to differential mechanical and evaporative stress with the association of their virulent genes in fomite-mediated environmental persistence and intra-host pathogenesis, which could be extended to investigate other bacterial pathogens as well.

### Limitations of the study

The mechanical characterization has brought out a global picture of impact dynamics of bacteria-laden droplets. However, sophisticated experimental techniques are needed to investigate the local effects of bacterial-laden droplets specially to study the variations of interferometric patterns. Furthermore, the study is limited to impact dynamics over a glass substrate and hence need to be investigated for surfaces with different properties. In addition, the regulation of bacterial gene network under velocity-induced mechanical stress and desiccation stress could have been studied in great details. In the current study, the bacteria recovered from the desiccated droplets (with or without impact velocity) were used for macrophage infection. The expression of bacterial genes associated with virulence was quantified from infected macrophages. Similar kind of gene expression study should be done after the recovery of the bacteria from the dried droplets before using them for infection. In the current study, the significant reduction in the *in vitro* viability of bacteria because of mechanical and desiccation stress was a major obstacle in direct isolation of RNA from the dried droplets.

## Ethical statement

The Institutional Animal Ethics Committee (IAEC) at Indian Institute of Science, Bangalore, India (Registration No: 48/1999/CPCSEA) approved all the animal experiment. The guidelines of CPCSEA were followed while conducting the animal experiments. The Committee for the Purpose of Control and Supervision of Experiments on Animals (CPCSEA) was formed under Chapter 4, Section 15(1) of the Prevention of Cruelty to Animals Act 1960. Ethical clearance number used to conduct the study is CAF/Ethics/670/2019.

## STAR★Methods

### Key resources table

**Table:** 

REAGENT or RESOURCE	SOURCE	IDENTIFIER
**Bacterial and virus strains**		

*Salmonella enterica* serovar Typhimurium ATCC strain14028S	*Gifted by Prof. M. Hensel*	Wild-type (WT)
*S.* Typhimurium *ΔphoP*	*Laboratory stock*	Chl^R^

**Chemicals, peptides, and recombinant proteins**		

Luria-Bertani broth	HIMEDIA	M575-500G
D-Glucose	*Qualigens*	Q15405
Mucin	Sigma-Aldrich	M1778-100G
*Salmonella-Shigella* agar	HIMEDIA	M108-500G

### Resource availability

#### Lead contact

Any information and request related to reagents and resources should be directed to Prof. Saptarshi Basu (sbasu@iisc.ac.in).

#### Materials availability

This study didn’t generate any new or unique resource or reagent.

### Method details

Figure 2A depicts the pictorial representation of the instrumentation for measuring impact velocity, spreading, receding diameters, and the impact droplet deposition patterns captured using side-view imaging, fluorescence microscopy, and reflection interference microscopy. Further, the *in vitro* bacterial viability after evaporation (environmental persistence), the infectivity of the bacteria recovered from the impacted dried droplets (in cell line and mouse infection model), and the effect of mechanical stress on the expression of bacterial genes during infection (by RNA isolation, cDNA synthesis and RT-qPCR method) were studied, as depicted in Figure 2B.

#### Preparation of bacterial samples

Bacterial solutions were prepared by modifying a protocol as demonstrated earlier.^4^ Briefly, overnight grown (10–12 hours old) stationary-phase cultures of STM (WT) and *ΔphoP* (corresponding to 10^8^ CFU/mL) were centrifuged at 5000 rpm for 10 minutes. The bacterial pellets were washed twice with double autoclaved milli-Q water and finally resuspended in 5 mL of milli-Q (neutral), 5% glucose (low-nutrient), 10% glycerol (low-nutrient), LB broth (nutrient-rich), 0.1 and 0.6 wt.% mucin (nutrient-rich) solutions to prepare the base medium.

#### Side-view visualization

The LED light source (3W) is aligned in the direction of the *Phantom Miro 110* camera to capture the drop impact phenomena for varying impact velocities. The images have been recorded at 10000 frames per second to measure droplet impact velocities and in-situ visualization of the droplet. The droplet impact velocities considered in this study (5, 7, and 10 m/s) were verified using this technique. The uncertainty of impact velocities is in the range of ±0.5 m/s.

#### Interference imaging

The plain glass slides (Bluestar©) kept in propan-2-ol, sonicated for 10 minutes, followed by rinsing with Kimwipes (Kimberly Clark International), were used to impact the bacterial-laden droplet at different velocities. The bacteria-laden droplet spreading dynamics were captured using the reflection interference microscope with interferometric optics, including a *Navitar* zoom lens, dichroic mirror, and beam splitter, and a microscope objective (5×,10×) aligned for capturing the impact of a droplet on the glass surface for varying impact velocities from the bottom view. A laser light source of wavelength 640 nm (*Cavitar, Cavilux smart UHS, 400W power*) is in-line alignment with a beam splitter. An objective lens is placed at its focal length between the glass slide and the beam splitter for the fine focus of the light beam. The reflected beam from the beam splitter passes through the bottom of the glass surface, and then the reflected light travels through the beam splitter to develop an interference fringe pattern, which is captured using high-speed imaging systems. The spreading dynamics of the drop impact have been recorded at 10000 frames per second (*Photron, SA5 camera*) simultaneously with the side-view imaging.

#### Atomic Force Microscopy

The dried precipitate patterns over the plain glass slides are viewed under a bio-Atomic Force Microscopy (AFM) (*Park System, South Korea*) integrated with an optical microscope with an X-Y flat scanner to observe the bacterial physiology. The scanner is used in both contact and non-contact modes to acquire microscopic, scanned images, adhesion energy, and variation of force-distance spectroscopy data for different samples. The XEI, XEP software is integrated with the instrument used to operate, analyze, and store data to estimate various mechanical parameters. The technique of AFM measurement is based on laser beam deflection, where the laser beam is reflected from the rear side of the cantilever onto a position-sensitive detector. The position and force given to the sample are regulated using the instrumental software. ACTA cantilever with high stiffness and the resonance frequency is used to get the topographical images of the sample. The contact mode AFM is carried out using CONTSCR (256px, scan rate 0.5 Hz) with less than an 8 nm scanning radius. The stiffness of the cantilever is 0.2 N/m with a resonance frequency of 25 kHz for acquiring the force indentation curve. Cantilever sensitivity and the spring constant are calibrated before each experiment runs.

#### Atomic force microscopy analysis

The initial AFM data acquired from the bacterial sample is analyzed using XEI software provided by Park System. The force-indentation curves were processed for baseline correction to nullify the cantilever-bending to accurately determine the tip-sample contact point and indentation force. The roughness was calculated by averaging the RMS roughness of three independent 5 μm^2^ areas.

#### Assessment of the i*n vitro* viability of the bacteria

The *in vitro* viability of the bacteria was measured by following a protocol as demonstrated earlier.^4^ 0.5 μL of the bacterial samples, prepared in different base mediums, were dispensed on a sterile glass slide with the help of a micropipette. The bacteria droplet is impacted at three different velocities on the glass substrate using a *PICO Pμlse* micro dispensing system (*Nordson, USA*) by applying suitable fluid pressure to achieve the desired velocity (5,7, and 10 m/s). The experiments are conducted at room temperature 25°C and relative humidity of 45%. Five hours after the desiccation, 100 μL sterile PBS was added to the top of the slide to resuspend the dried bacterial droplets of different solutions. After resuspension, this 100 μL PBS was directly plated on *Salmonella*-*Shigella* (SS) agar. 100 μL from the overnight-grown culture and freshly prepared bacterial suspensions were plated to calculate the bacterial count in 0.5 μL of planktonic culture. After incubating the plates for sixteen hours at 37°C temperature, the viable bacterial load was enumerated.

#### Infection of RAW264.7 macrophages

The infection of the RAW264.7 macrophages was done following a protocol as demonstrated earlier.^4^ Wild-type *Salmonella* Typhimurium was retrieved from the desiccated dried droplet of mucin as described earlier and used to infect RAW264.7 macrophage cells. The infected cells were incubated in a 5% CO_2_ incubator for 30 minutes at 37°C temperature, followed by washing with sterile PBS to clear the unattached bacteria. The cells were further incubated with 100 μg/mL of gentamycin for an hour and 25 μg/mL of gentamycin till the end of the experiment. The cells were lysed with 0.1% Triton-X100 at 3 hours and 18 hours. The lysates were serially diluted with PBS and plated on SS agar. To calculate the bacterial fold proliferation, the CFU was obtained from 18hr. was divided with the CFU from 3 hr. The CFU obtained from 3 hr. was further divided with the pre-inoculum CFU to calculate the percent phagocytosis of macrophages.

#### Isolation of RNA from infected macrophages

The isolation of RNA from infected macrophages was performed using a protocol as demonstrated earlier.^71^^,^^72^ The infected macrophage cells were treated with 1 mL of TRIzol solutions for lysis and kept at −80°C overnight. The lysates were further subjected to chloroform extraction, and to the isolated aqueous layer, an equal volume of isopropanol was added. The RNA was precipitated by centrifuging the mixture of an aqueous layer and isopropanol at 14000 rpm for 20 minutes. The RNA pellet was washed with 70% ethanol and resuspended in DEPC water. The RNA was treated with RNase-free DNase and converted into cDNA with the PrimeScript^TM^ cDNA synthesis kit. The cDNA was subjected to RT-qPCR using SYBR green RT-qPCR kit to estimate the transcript-level expression of *sifA*, *ssaV*, and *phoP*.

#### Determining the bacterial burden from the organs of C57BL/6 mice

The bacterial load from the organs of infected animals was determined by following a protocol demonstrated earlier.^22^^,^^71^^,^^73^ Wild-type *Salmonella* Typhimurium was retrieved from the desiccated dried droplet of mucin (0.1 wt.%) as described earlier^4^ and used to infect 4–6 weeksold C57BL/6 mice. 10^6^ CFU of overnight grown stationary phase planktonic culture of *Salmonella* was used as control. On the 6^th^ day post-infection, the animals were sacrificed, and the liver and spleen were collected. The infected organs were homogenized, and the lysates were serially diluted for plating on *Salmonella-Shigella* agar. The CFU obtained from each organ was normalized with the weight of the tissues used from homogenization.

### Quantification and statistical analysis

Each experiment has been independently repeated several times, as indicated in the figure legends (n = technical replicates and N = biological replicates). GraphPad Prism 8.4.3 software was used to generate the graphs and perform the statistical tests. All statistics were performed in the manuscript by unpaired students t-test, as mentioned in the figure legends. The *p*-values below 0.05 were considered significant in all the studies.

## Data Availability

• This study didn’t generate datasets/codes. • All relevant data are within the paper and its Supporting Information files. • Further information related to the data is available upon request to the lead contact, Prof. Saptarshi Basu (sbasu@iisc.ac.in).
