# Supplementary material for: phoP maintains the environmental persistence and virulence of pathogenic bacteria in mechanically stressed desiccated droplets
Source: iScience. 2023 Apr 8;26(5):106580. doi: 10.1016/j.isci.2023.106580 (PMC10164896; doi:10.1016/j.isci.2023.106580)
Supplement: Document S1. Figures S1–S5 [file mmc1.pdf]

iScience, Volume *26*

Supplemental information

phoP maintains the environmental persistence and virulence of pathogenic bacteria in mechanically stressed desiccated droplets

Vishnu Hariharan, Atish Roy Chowdhury, Srinivas Rao S, Dipshikha Chakravortty, and Saptarshi Basu

Supplementary materials

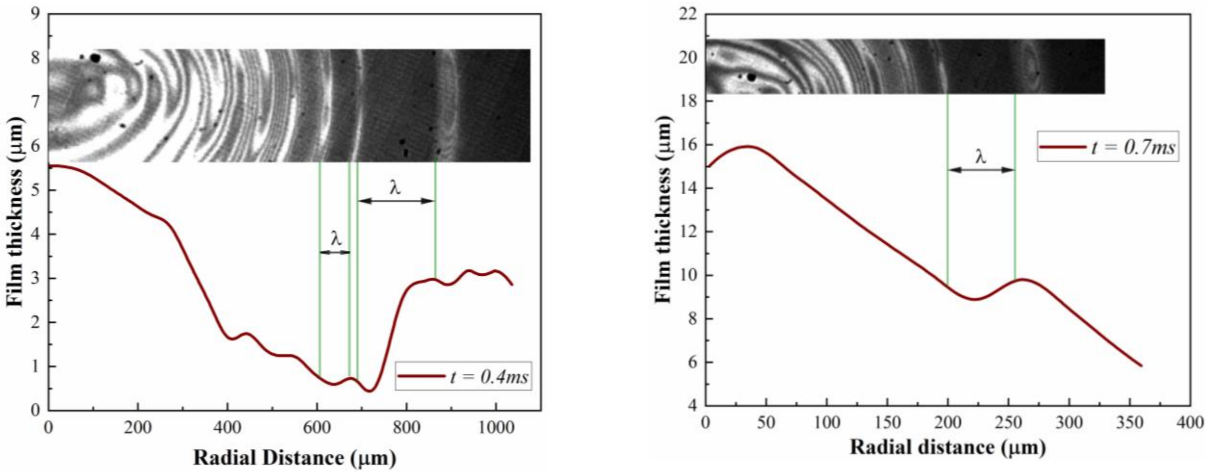

Figure S1 Variation of thin-film layer thickness and capillary wavelengths

Variation of film-thickness for time instants from 0.3 to 3.8 ms and Film thickness and observance of capillary waves at time instants of 0.4 and 0.7 ms. (Relates to **Figure 2B**)

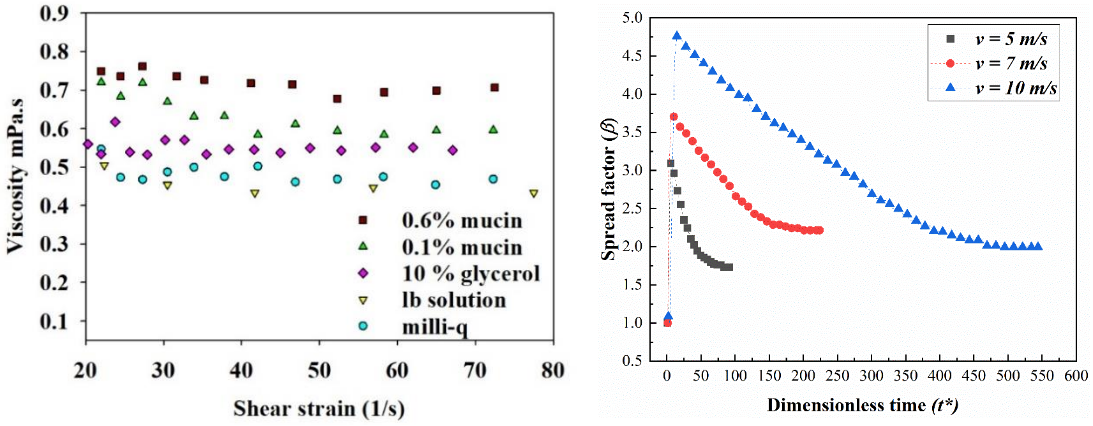

Figure S2 Fluid medium properties for different impact velocities

Viscosity comparison for different samples milli-Q, 10% glycerol, LB solution, 0.6 wt.%, and 0.1 wt.% mucins (left) and Viscosity comparison of different fluids and spread factor comparison at different impact conditions (right). (Relates to **Figure 2A**)

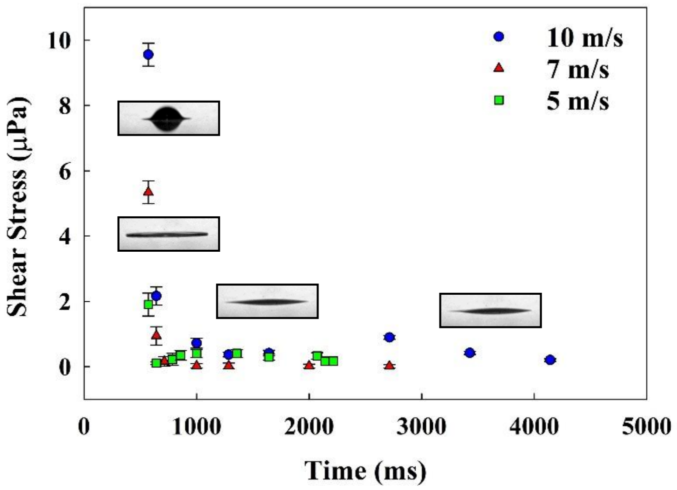

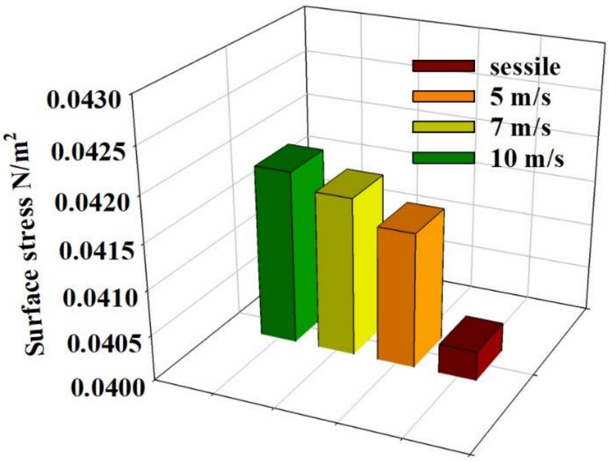

Figure S3 Shear stress variation and surface stress experienced by AFM probe.

The shear stress variation for the impacted sample on glass substrate (top) and surface stress experienced by the AFM probe on four different impact conditions with milli-Q as the base medium (bottom). (Relates to **Figure 2A, 2B, 2C, 2D**)

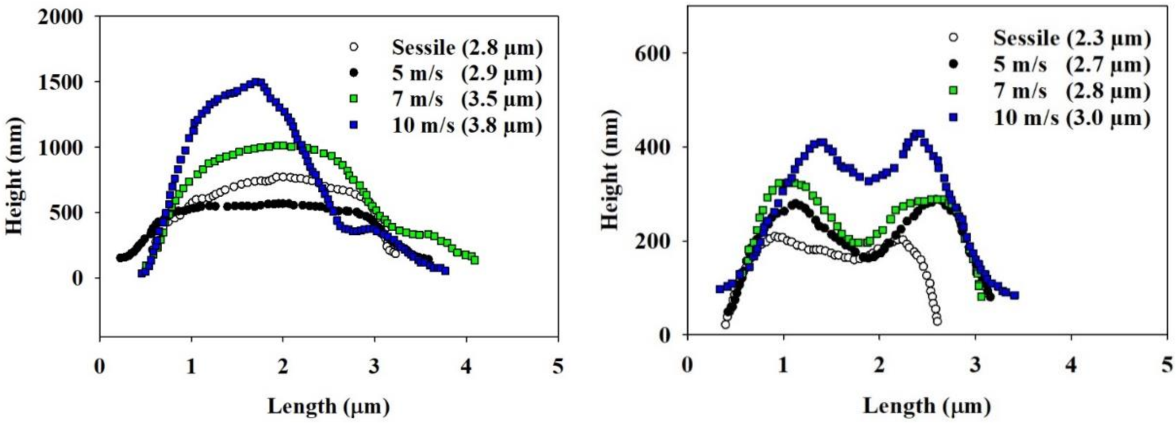

Figure S4 Height and length profile of a random bacteria under different impact conditions.

The height profile of *Salmonella* Typhimurium in milli-Q (left) and 0.6% mucin (right) subjected to different impact conditions. (Relates to **Figure 2E, 2F, 2H**)

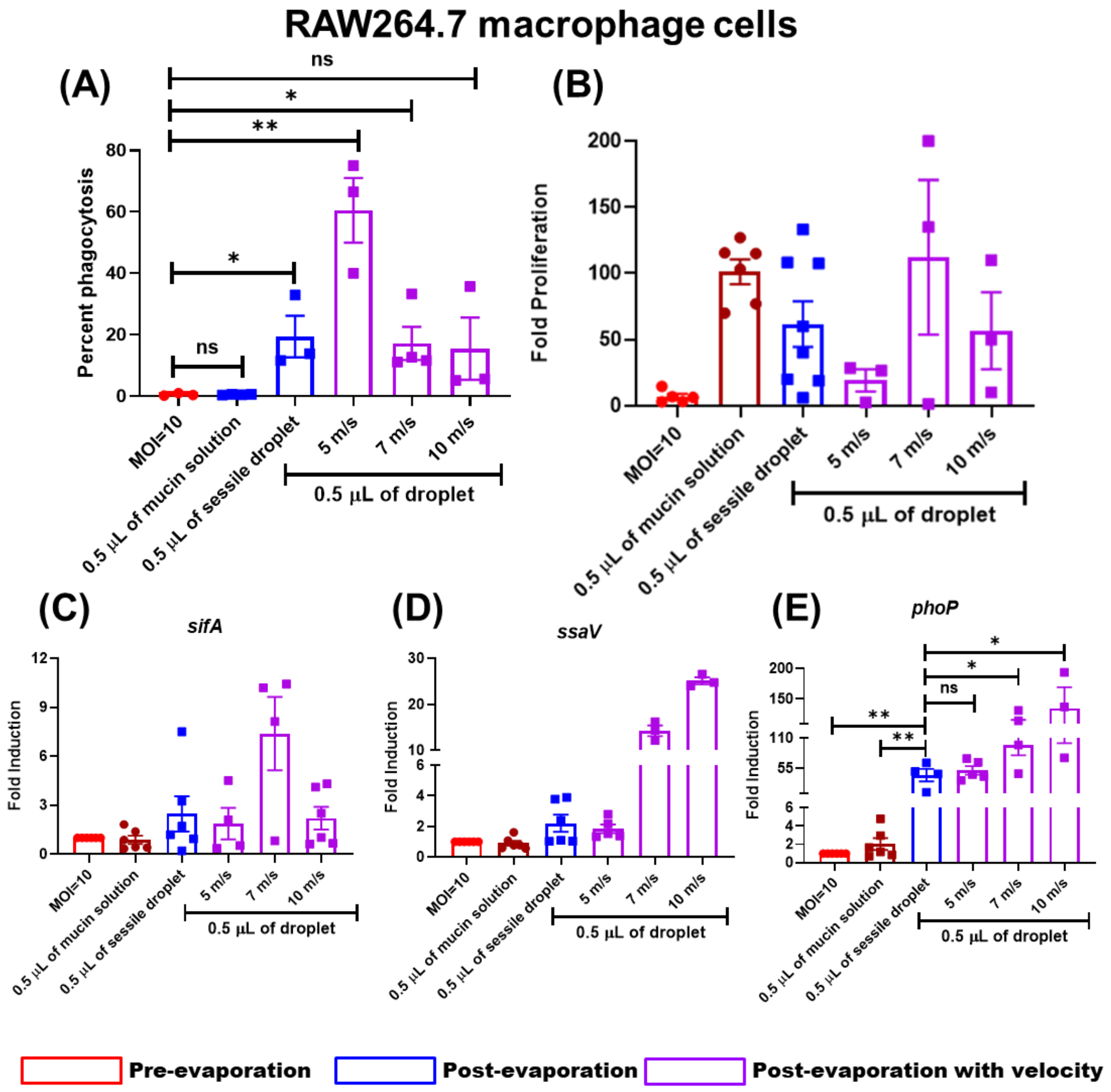

Figure S5 The virulence of *Salmonella* Typhimurium retrieved from desiccated mucin droplet (0.6 wt%) impacted solid surface with or without velocities.

Salmonella Typhimurium recovered from desiccated mucin (0.6 wt%) droplet impacted solid glass surface with or without specific velocities (5, 7, and 10 m/s) were used to infect RAW264.7 cells to determine the
(A) percent phagocytosis and **(B)** intracellular proliferation (n=3, N=2).
Determining the transcript-level expression of **(C)** *sifA*, **(D)** *ssaV* and **(E)** *phoP* from intracellular *Salmonella* by RT-qPCR (n=3, N=2).
Data are represented as mean ± SEM. . (Relates to **Figure 4A-4E**)

(P)* *< 0.05, *(P)* **< 0.005, *(P)* ***< 0.0005, *(P)* *< 0.0001, ns= non-significant, (Student's t test-unpaired)**
